# Use of a design of experiments approach to optimise production of a recombinant antibody fragment in the periplasm of *Escherichia coli:* selection of signal peptide and optimal growth conditions

**DOI:** 10.1186/s13568-018-0727-8

**Published:** 2019-01-07

**Authors:** Ikhlaas M. Kasli, Owen R. T. Thomas, Tim W. Overton

**Affiliations:** 1 School of Chemical Engineering, University of Birmingham, Edgbaston, Birmingham, B15 2TT UK; 2 Institute of Microbiology and Infection, University of Birmingham, Edgbaston, Birmingham, B15 2TT UK

**Keywords:** Fermentation, Heterologous protein, Biopharmaceutical, Single-chain variable fragment (scFv)

## Abstract

Production of recombinant proteins such as antibody fragments in the periplasm of the bacterium *Escherichia coli* has a number of advantages, including the ability to form disulphide bonds, aiding correct folding, and the relative ease of release and subsequent capture and purification. In this study, we employed two N-terminal signal peptides, PelB and DsbA, to direct a recombinant scFv antibody (single-chain variable fragment), 13R4, to the periplasm via the Sec and SRP pathways respectively. A design of experiments (DoE) approach was used to optimise process conditions (temperature, inducer concentration and induction point) influencing bacterial physiology and the productivity, solubility and location of scFv. The DoE study indicated that titre and subcellular location of the scFv depend on the temperature and inducer concentration employed, and also revealed the superiority of the PelB signal peptide over the DsbA signal peptide in terms of scFv solubility and cell physiology. Baffled shake flasks were subsequently used to optimise scFv production at higher biomass concentrations. Conditions that minimised stress (low temperature) were shown to be beneficial to production of periplasmic scFv. This study highlights the importance of signal peptide selection and process optimisation for the production of scFv antibodies, and demonstrates the utility of DoE for selection of optimal process parameters.

**Electronic supplementary material:**

The online version of this article (10.1186/s13568-018-0727-8) contains supplementary material, which is available to authorized users.

## Introduction

Recombinant protein production (RPP) is an industrially important tool for the production of hundreds of licensed recombinant proteins (RPs), including IgG antibodies and antibody fragments (Walsh 2014; Sanchez-Garcia et al. 2016). Unlike their larger full-length IgG monoclonal antibody counterparts, which are commonly produced in mammalian cells, the relative simplicity of antibody fragments and their requirement for fewer post-translational modifications makes them suitable for production in bacterial hosts. The bacterium *Escherichia coli* is a commonly employed host for recombinant protein production (RPP) contributing to the production of one-third of FDA approved human biotherapeutics (Overton 2014; Walsh 2014). Single chain variable fragments (scFv) are an emerging class of IgG fragments comprising the antigen-binding variable heavy (V_H_) and variable light (V_L_) domains fused into a single polypeptide chain with a flexible linker (Nelson 2010).

The suitability of *E. coli* as a host for production of recombinant antibody fragments and other human biotherapeutic proteins stems, in large part from the following: (i) its physiology, metabolism and behaviour are very well understood compared to other bacterial species; (ii) it exhibits much faster growth, attains higher cell densities and also requires much cheaper growth media than mammalian hosts; and (iii) although it cannot produce RPs with ‘human-like’ glycosylation, it can generate disulphide bonds (Plückthun and Skerra 1989; Hsu et al. 2016).

Unlike the cytoplasm, which is a reducing environment, the periplasm is an oxidising environment and so favours the formation of disulphide bonds (de Marco 2009); it also contains enzymes which catalyse the formation, correction and maintenance of disulphide bonds (the Dsb enzymes (Inaba 2009). The periplasm of *E. coli* offers additional advantages as a cellular compartment for targeting RPs in bioprocesses. It contains fewer proteases than the cytoplasm, which reduces the risk of proteolytic degradation during growth; and accounts for just 4–8% of the *E. coli* protein content (Beacham 1979). Moreover, a periplasmic location affords selective extraction using approaches that disrupt or destabilise the outer membrane and cell wall, but not inner membrane (Neu and Heppel 1965; Katsui et al. 1982; Naglak and Wang 1990; Weir and Bailey 1995; Kraemer et al. 2016) thereby reducing demands on subsequent purification.

*Escherichia coli* possesses three major mechanisms for the transport of proteins from the cytoplasm to the periplasm. Two of these, the general secretory (Sec) and signal recognition particle (SRP) pathways, direct unfolded polypeptide chains through a protein pore in the inner membrane (the SecYEG complex), one amino acid at a time (Natale et al. 2008; Du Plessis et al. 2011; Tsirigotaki et al. 2017). In the Sec or ‘post-translational’ pathway, the polypeptide chain is translocated following complete translation by the ribosome. In the SRP or ‘co-translational’ pathway, the polypeptide chain is bound by the SRP and translocated by SecYEG whilst it is still being translated. In each case, an N-terminal signal peptide directs the polypeptide chain to the correct pathway (Freudl 2018).

During RPP processes, a key need is balancing the energy and metabolite requirements of biomass generation and RP synthesis; failure to do this has a detrimental impact on the host cell physiology, and also impairs the solubility, folding and resultant functionality of the RP (Villaverde and Carrió 2003). One common approach is to temporally segregate the biomass-production and RPP phases by employing a tightly-regulated promoter controlling RP synthesis, in combination with a chemical inducer (Overton 2014). An alternate approach is ‘stress minimisation’, whereby growth and RPP proceed concurrently, albeit at a slower rates. Practically, this is achieved by adopting lower growth temperatures and low inducer concentrations. In this way RP is translated more slowly enabling in turn more efficient folding and therefore a higher proportion of soluble, functional RP (Sevastsyanovich et al. 2009). Stress minimisation approaches have been successfully used to optimise production of cytoplasmic model proteins (Sevastsyanovich et al. 2009; Wyre and Overton 2014), cytoplasmic biotherapeutics (Selas Castiñeiras et al. 2018b) and periplasmically-targeted antibody fragments (Hsu et al. 2016; Selas Castiñeiras et al. 2018a) in fed-batch fermentation processes.

In this study a design of experiments (DoE) approach with a three-factor central composite design was used to optimise and characterise the design space for the production of a model scFv, 13R4 (Martineau et al. 1998) targeted to the periplasm of *E. coli* via either the SRP or SecB pathway. The use of a central composite design allowed for the minimisation of the number of cultures whilst permitting statistical analysis. The factors varied were fermentation temperature, concentration of inducer (arabinose) and the OD_600_ at which induction occurred; three factors known to be important in RPP (Sevastsyanovich et al. 2009; Wyre and Overton 2014; Hsu et al. 2016; Selas Castiñeiras et al. 2018b). The responses measured were the productivity, solubility and location of scFv 13R4, and measures of bacterial physiology, (Nebe-Von-Caron et al. 2000; Wyre and Overton 2014); biomass concentration (from optical density measurements); and culturability, determined using colony forming unit (CFU) counts. Models generated by this DoE approach were used to inform the design of baffled shake flask experiments, supporting higher biomass concentrations. We show that conditions that minimise stress (low temperature and inducer concentration) are favourable for production of soluble, periplasmic scFv, and demonstrate the utility of the DoE approach in identifying the optimum operation window coinciding with minimum stress conditions.

## Materials and methods

### Strains and plasmids

*Escherichia coli* strain BL21 (*F*^−^ *ompT gal dcm lon hsdS*_*B*_ (*r*_*B*_^−^*m*_*B*_^−^) *[malB*^+^*] K*-*12(λS) Δ(ara)*) was employed in all experiments. The plasmids used, pLBAD2-DsbA-scFv13R4+A and pLBAD2-PelB-scFv13R4+A (Selas Castiñeiras et al. 2018a), were sourced from Cobra Biologics; and encode scFv 13R4 (Martineau et al. 1998) directed to the periplasm by the *E. coli* DsbA and *Pectobacterium carotovorum* PelB signal peptides respectively, under the control of the *E. coli araBAD* promoter (Guzman et al. 1995). Both plasmids had the pMB1 origin of replication and encoded resistance to kanamycin.

### Shake flask cultures

Overnight cultures in test tubes containing 5 mL of Luria broth (Miller, Sigma-Aldrich item L3522, UK) supplemented with 50 µg mL^−1^ kanamycin were inoculated with a single colony of transformed *E. coli* and grown for 18 h at 30 °C and 150 rpm. One millilitre of this overnight culture was used to inoculate 100 mL of Terrific Broth containing 1.2% (w/v) tryptone (Oxoid, UK), 2.4% (w/v) yeast extract (Oxoid, UK), 0.4% (v/v) glycerol, 16.9 mM KH_2_PO_4_ and 71.8 mM K_2_HPO_4_ supplemented with 50 µg mL^−1^ kanamycin in a 250 mL conical flask or 500 mL baffled shake flask. Shake flask cultures were grown and induced according to the conditions defined by the DoE and described in Additional file 1: Table S1, with agitation at 150 rpm. Samples of each culture were taken at time intervals for analysis. Cell pellets from [0.9/OD_600_] mL of culture were stored at − 20 °C until fractionation and/or SDS-PAGE.

### Spectrophotometry and flow cytometry

The optical density of cultures was measured at 600 nm (OD_600_) using an Evolution 200 Spectrophotometer (ThermoFisher Scientific, UK). Cultures were analysed using a BD Accuri C6 flow cytometer (Becton–Dickinson, UK); bacteria were diluted in 1 mL of 0.22 µm-filtered phosphate buffered saline (PBS; Oxoid, UK) and incubated with 4 µg mL^−1^ propidium iodide (PI; Sigma-Aldrich, UK) and 0.1 µg mL^−1^ bis-(1,3-dibutylbarbituric acid) trimethineoxonol (BOX; ThermoFisher Scientific, UK) for 5 min before analysis. Data were collected at a rate of 1000–4000 events per second using a forward scatter height (FSC-H) threshold of 12,000 until 25,000 events had been recorded. PI and BOX fluorescence were measured via channels FL1-A (533/30 filter) and FL3-A (670 LP filter) respectively. Dead cell controls for PI and BOX were prepared by incubating a culture at 99 °C for 10 min prior to analysis.

### Colony forming units and replica plating

Bacterial cultures were serially diluted in sterile PBS (Oxoid, UK); the two most appropriate dilutions were plated onto nutrient agar (Oxoid, UK) plates and incubated at 30 °C for 18 h before the colonies were counted. The plate containing closest to 300 colonies was stamped with sterile filter paper (Fisherbrand, USA) and stamped onto nutrient agar plates supplemented with and without 50 µg mL^−1^ kanamycin. After incubation at 30 °C for 18 h, colonies were counted and the percentage that had retained the plasmid was calculated.

### Fractionation of soluble and insoluble proteins

To normalise the quantity of biomass for each sample, bacteria from [0.9/OD_600_] mL of culture were collected by centrifugation (775*g*_av_, 5 min) and resuspended in 0.25 mL of PBS (pH 8) containing 0.02% (w/v) lysozyme (ThermoFisher Scientific, UK) and 0.4% (v/v) benzonase (Sigma-Aldrich, UK). The suspensions were incubated on ice with gentle shaking for 30–45 min, then frozen in a dry ice and ethanol bath and thawed at 30 °C a minimum of three times, then centrifuged at 9000*g*_av_ for 30 min. The supernatant (soluble fraction) was separated from the pellet (insoluble fraction), which was resuspended in 0.25 mL of PBS (pH 8); both fractions were stored at − 20 °C until analysis by SDS-PAGE.

### Fractionation of periplasmic and spheroplast proteins

Bacteria from [0.9/OD_600_] mL of culture were collected by centrifugation (775*g*_av_, 5 min), resuspended in 100 µL of ice cold spheroplast buffer (100 mM Tris-HCl pH 8, 500 mM sucrose, 0.5 mM EDTA) and incubated on ice for 5 min. The cell suspensions were centrifuged at 14,000*g*_av_ for 1 min, and the supernatant discarded. The pellet was resuspended by vigorous pipetting in 95 µL of ice cold 0.22 µm-filter sterilised water. After 30 s, 5 µL of 20 mM MgCl_2_ was added and the suspensions centrifuged at 14,000*g*_av_ for 2 min. The supernatant (periplasmic fraction) was separated from pellet (spheroplast fraction). The pellet was resuspended in 100 µL of ice cold spheroplast buffer and both fractions were stored at − 20 °C until analysis by SDS-PAGE. Prior to preparation for SDS-PAGE, spheroplast fractions were incubated at 99 °C for 10 min with shaking at 500 rpm.

### Sodium dodecyl sulphate polyacrylamide gel electrophoresis (SDS-PAGE) and scanning densitometry

Protein compositions of fractions were analysed by reducing SDS-PAGE (Laemmli 1970) in Tris-Glycine SDS precast 12% (w/v) polyacrylamide gels (Novex WedgeWell, ThermoFisher Scientific, UK) in a Mini Gel Tank (ThermoFisher Scientific, UK) system at 200 V for 45 min. Samples containing protein were mixed with 40 µL of Tris-Glycine SDS sample buffer (63 mM Tris HCl pH 6.8, 10% (v/v) glycerol, 2% (w/v) SDS, 0.0025% (w/v) bromophenol blue), 8 μL of 500 mM DL-dithiothreitol (DTT) and 12 μL of deionised water. All samples were incubated at 85 °C for 2 min prior to loading onto the gel. Spheroplast samples were mixed with Tris-Glycine SDS sample buffer and incubated at 99 °C for 10 min before the DTT was added.

All gels were calibrated for molecular weight by loading free wells with 5 µL of an 11 to 190 kDa ladder of Blue Prestained Protein Standards (New England BioLabs, USA) of known concentration (0.2 mg/mL). After electrophoresis protein bands in gels were visualised using SimplyBlue™ Safe Stain (ThermoFisher Scientific, UK), imaged on a flatbed scanner CanoScan 9000F, Canon, UK) at a resolution of 4800 dpi, and subsequently analysed densitometrically using ImageJ software (Schneider et al. 2012) downloaded from http://rsb.infonih.gov.ij/. Productivity of scFv was determined by comparison the intensity of scFv bands to the intensity of the 25 kDa band in the 11–190 kDa reference ladder of known protein concentration. This value was then divided by the fermentation running time and calculated to give the productivity in microgram of recombinant scFv per millilitre of culture broth per hour of growth.

### Design of experiments and data treatment

The design of experiment (DoE) protocol was designed in Design-Expert version 7.1 (StatEase). The design created was a circumscribed three factor, 5-level central composite design with 10 repeats; with each factor at level ‘0’, resulting in 24 runs. Once data had been collected they were checked for normality using a normal probability plot so as to ensure suitability for parametric statistical testing. The PelB productivity data were normalised by a square root transformation. The models produced were subsequently checked for significance and insignificant lack of fit. Models were used to produce contour plots, with each showing the response while two factors varied, and the third factor was maintained at level ‘0’.

### RNA structure prediction

The structure of the first 100 bases of RNA encoding PelB^sp^-scFv and DsbA^sp^-scFv was predicted using the RNAfold Webserver (Lorenz et al. 2011).

## Results

A DoE approach using a central composite design was employed to optimise the production of the model scFv 13R4 (Martineau et al. 1998), henceforth referred to as scFv. Two plasmids were used to target scFv to the periplasm, one using the *E. coli* DsbA signal peptide, DsbA^sp^ (Schierle et al. 2003) and the SRP pathway, the other employing *Pectobacterium carotovorum* PelB signal peptide, PelB^sp^ (Lei et al. 1987) and the SecB pathway. The arabinose-inducible pBAD promoter (Guzman et al. 1995) regulated scFv expression from both plasmids. The variables in these experiments were: temperature (20.6 °C to 39.9 °C); the concentration of arabinose used to induce recombinant protein production (0 to 0.26% w/v, the pBAD user manual suggests a maximum arabinose concentration of 0.2% (Invitrogen 2010); and the OD_600_ at which induction occurred, between 0.29 (early exponential) and 1.21 (early stationary phase). Levels for each variable are shown in Table 1. Preliminary experiments confirmed that these ranges were suitable for these strains and growth conditions. Previous studies have identified that: (i) lower temperatures are preferable for correct folding of RPs (Vera et al. 2007), though very slow growth is observed below 20 °C; (ii) temperatures above 40 °C illicit induction of the heat shock response, potentially increasing proteolysis (Meyer and Baker 2011); and (iii) a temperature of 25 °C is a good compromise between growth and RP folding (Sevastsyanovich et al. 2009). All cultures were grown in 100 mL of terrific broth with 0.4% (w/v) glycerol as a carbon source in 250 mL conical flasks. Additional file 1: Table S1 lists the growth conditions of each individual culture.

**Table 1:** Levels for variables in the central composite design experiments

Variable	Level				
	− α	− 1	0	+ 1	+ α
Temperature (°C)	30.6	25.0	30.3	35.5	39.9
OD_600_ at induction	0.29	0.50	0.75	1.00	1.21
[Arabinose] (% w/v)	0.000	0.059	0.130	0.200	0.259

Samples were analysed on induction and 4 h and 6 h after induction. Responses measured were: overall scFv productivity (µg scFv mL^−1^ culture h^−1^, determined by SDS-PAGE); percentage of scFv that was soluble (determined by SDS-PAGE analysis of soluble and insoluble cell fractions); percentage of scFv in the periplasmic fraction (SDS-PAGE analysis of cytoplasmic and periplasmic fractions); percentage of “healthy” cells (determined by flow cytometry and defined as having both membrane potential and membrane integrity thereby staining with neither PI nor BOX); CFU mL^−1^; and percentage plasmid retention (measured by replica plating). In both the DsbA^sp^-scFv and PelB^sp^-scFv systems, the responses which produced significant (p < 0.05) models with a non-significant (p > 0.05) lack of fit (tested by ANOVA) were productivity and the percentage of scFv that was soluble; both for samples taken 6 h after induction (Additional file 1: Tables S2–S5). Most other models yielded significant lack of fit.

**Fig. 1:**
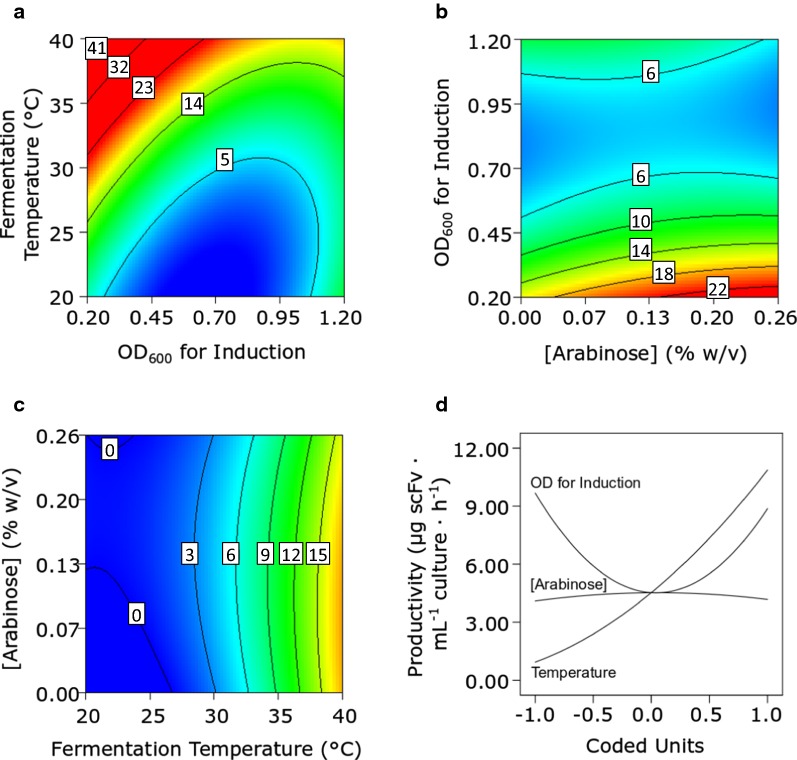
Response (**a**–**c**) and perturbation (**d**) plots for the whole cell productivity of DsbA^sp^-scFv. Response plots show the response of two factors; the third invariant factor is set at level ‘0’. Units in response plots are µg scFv mL^−1^ culture h^−1^

The overall scFv productivity response curves for DsbA^sp^-scFv are presented in Fig. 1. Raising the fermentation temperature alone increased scFv productivity almost tenfold (Fig. 1a). Temperature and induction OD_600_ also interacted to increase productivity; i.e. when a high fermentation temperature is coupled with a low induction OD_600_ (Fig. 1a) productivity exceeded > 40 µg scFv mL^−1^ h^−1^. Figure 1b and c respectively show that whereas scFv productivity rises when the induction OD_600_ is lowered, changes in the concentration of arabinose exert little effect on the scFv productivity. The small effect of changes in arabinose concentration can also be seen in the perturbation plot (Fig. 1d), which clearly highlights the key impact of growth temperature on scFv productivity.

**Fig. 2:**
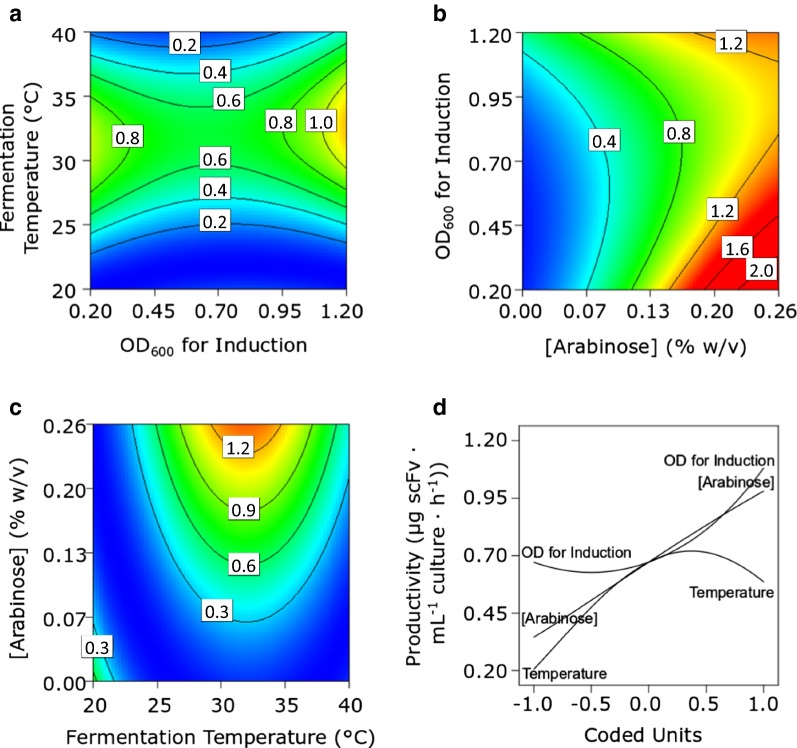
Response (**a**–**c**) and perturbation (**d**) plots for the whole cell productivity of PelB^sp^-scFv. Response plots show the response of two factors; the third invariant factor is set at level ‘0’. Units in response plots are µg scFv mL^1^ culture h^−1^

A very different pattern emerged from experiments with PelB^sp^-scFv (Fig. 2). A peak in overall scFv productivity in response to changing temperature was observed at around 32 °C (Fig. 2a). scFv productivity increased by more than fivefold when high concentrations of arabinose were used to induce the culture (Fig. 2b). Although this trend was observed regardless of the induction OD_600_, the strongest interaction was observed between early induction (at an OD_600_ of < 0.7) and a high inducer concentration, which generated the greatest increases in scFv productivity (> 2 µg scFv mL^−1^ culture h^−1^). Figure 2d shows an almost linear perturbation curve for arabinose concentration, which also creates the largest perturbation of all three factors. The optimal growth temperature of ≈ 32 °C is respectively defined by horizontal and vertical ridges in the ‘temperature vs. induction OD_600_’ (Fig. 2a) and ‘arabinose concentration vs. temperature’ (Fig. 2c) plots, and appears as a peak in the temperature perturbation curve (Fig. 2d). The induction OD_600_ perturbation curve (Fig. 2d) indicates that productivity is improved by inducing at higher cell densities, e.g. raising the induction OD_600_ from 0.625 to 1.21 led to a productivity increase of approximately 0.5 µg scFv mL^−1^ culture h^−1^. Overall, PelB^sp^-scFv productivity was far lower than that of DsbA^sp^-scFv.

**Fig. 3:**
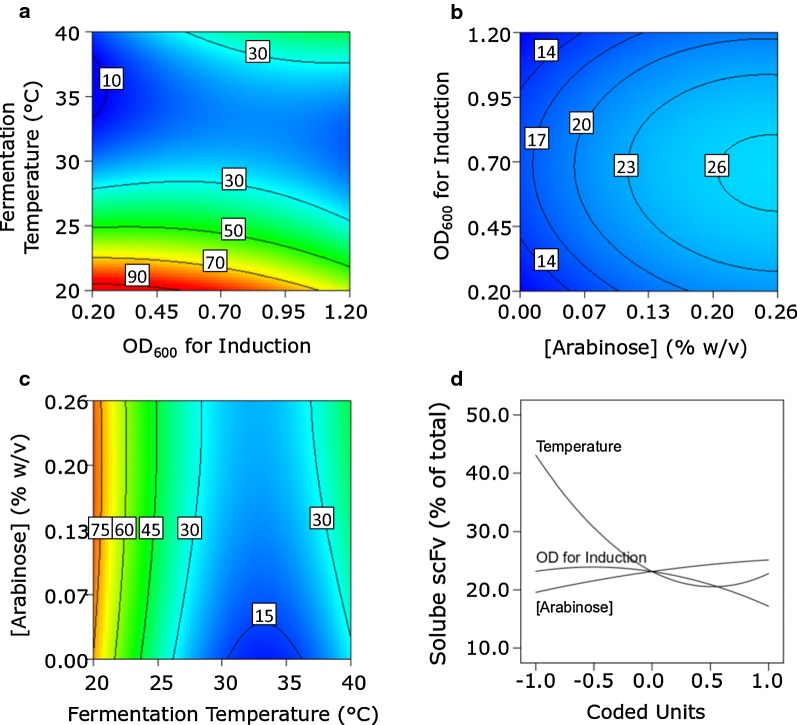
Response (**a**–**c**) and perturbation (**d**) plots for the percentage solubility of DsbA^sp^-scFv. Response plots show the response of two factors; the third invariant factor is set at level ‘0’. Units in response plots are µg scFv mL^−1^ culture h^−1^

**Fig. 4:**
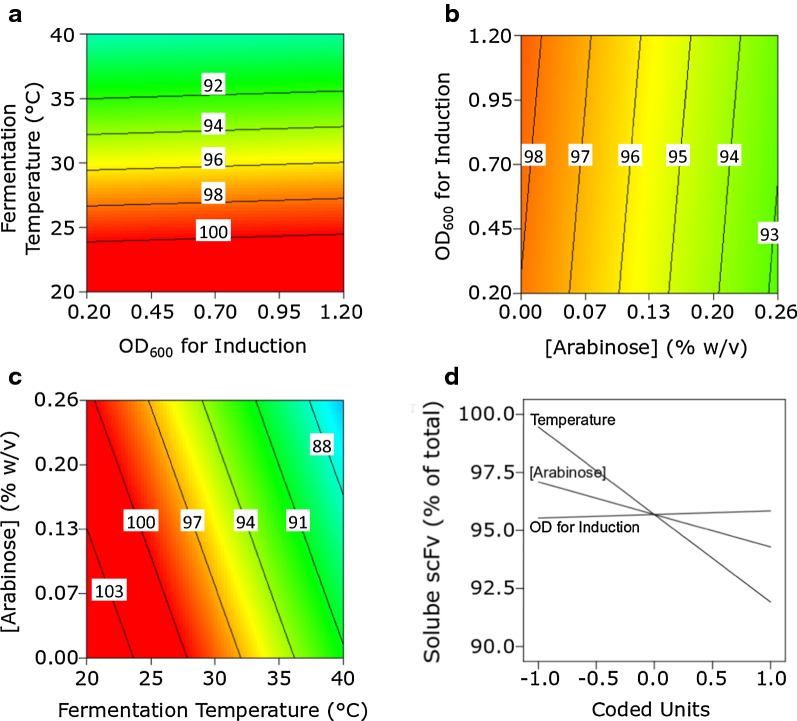
Response (**a**–**c**) and perturbation (**d**) plots for the percentage solubility of PelB^sp^-scFv. Response plots show the response of two factors; the third invariant factor is set at level ‘0’. Units in response plots are µg scFv mL^−1^ culture h^−1^

RP solubility is important in many bioprocesses, particularly when the RP in question cannot be readily refolded e.g. following solubilisation from insoluble inclusion bodies, or alternatively because protein refolding steps are ruled out on economic grounds (Overton 2014; Hoffmann et al. 2018). Figure 3 shows the response plots for soluble scFv expressed as a percentage of the total scFv produced by DsbA^sp^-scFv cultures. Reducing the fermentation temperature from 40 to 20 °C was accompanied by dramatic increases in the % scFv that was soluble (Fig. 3a, c). A very slight interaction with the induction OD_600_ and fermentation temperature was also noted, i.e. early induction coupled with low fermentation temperatures resulted in as much as 90% of the scFv in soluble form (Fig. 3a). Figure 3b shows part of a peak in the proportion of soluble scFv within the design space, observed at high arabinose concentration (> 0.2% w/v) with induction during the mid-logarithmic phase of growth (OD_600_ = 0.7). The maximum solubility in this contour plot is 26%, given that the third factor, temperature, is set to level ‘0’ (30.3 °C); Fig. 3a however indicates an increase in the proportion of soluble scFv with drop in temperature. The perturbation plot (Fig. 3d) reveals temperature to be the dominant influence on scFv solubility, with lower temperatures being advantageous to RP solubility, consistent with earlier observations (Sevastsyanovich et al. 2009; Wyre and Overton 2014); and also highlights the much lower solubility of DsbA^sp^-scFv compared to PelB^sp^-scFv (Fig. 4). As with DsbA^sp^-scFv, the largest influence on PelB^sp^-scFv solubility (Fig. 4a, c and d) came from temperature, i.e. lowering the growth temperature enhanced the scFv’s solubility, although PelB^sp^-scFv was inherently more soluble than DsbA^sp^-scFv in all growth conditions. scFv solubility was also influenced by changes in inducer concentration, solubility increasing with decreasing arabinose concentration. Maximum scFv solubility occurred at low temperature and low arabinose concentration, indicating an interaction between these two factors (Fig. 4c). By contrast, the impact of induction OD_600_ on scFv solubility was negligible (Fig. 4a, b and d).

In both systems, the fermentation temperature had a great effect on scFv productivity and also impacted on solubility, albeit to different degrees. Increasing the fermentation temperature from 20 to 40 °C was met with improved whole cell productivity in the case of DsbA^sp^-scFv, whereas optimal productivity was observed at ca. 32 °C for the PelB^sp^-scFv system. The observed enhancement in productivity at higher temperatures, likely due to faster rates of protein translocation and transcription, and faster growth leading to earlier induction, come at the expense of reduced scFv solubility.

**Fig. 5:**
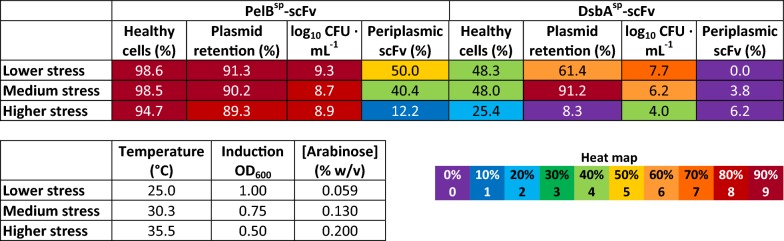
Heatmap of responses from the DsbA^sp^-scFv and PelB^sp^-scFv systems that did not produce significant models

Figure 5 shows data for the non-significant models as a heat map at three levels of stress, corresponding to the levels − 1/0/+ 1 for each factor (Fig. 5). For the PelB^sp^-scFv system, the proportion of “healthy” cells measured by flow cytometry (where PI^−^/BOX^−^ cells were considered healthy) and log_10_ CFU mL^−1^ were all higher than the DsbA^sp^-scFv system at all stress levels. Plasmid retention in PelB^sp^-scFv was similar to that in DsbA^sp^-scFv, or higher, whilst the percentage of scFv in the periplasm was greater for the PelB^sp^-scFv system.

In summary, DsbA^sp^ generated high scFv productivity, but the solubility and periplasmic targeting of scFv, and overall cell physiology (membrane potential, membrane integrity and culturability) were poor. Conversely scFv productivity from PelB^sp^ was much lower *cf.* DsbA^sp^, but scFv solubility and periplasmic targeting were all high; cell physiology was also better than for DsbA^sp^. For these reasons, PelB^sp^ was chosen as the signal peptide for further optimisation.

### Intensification to baffled shake flasks

**Fig. 6:**
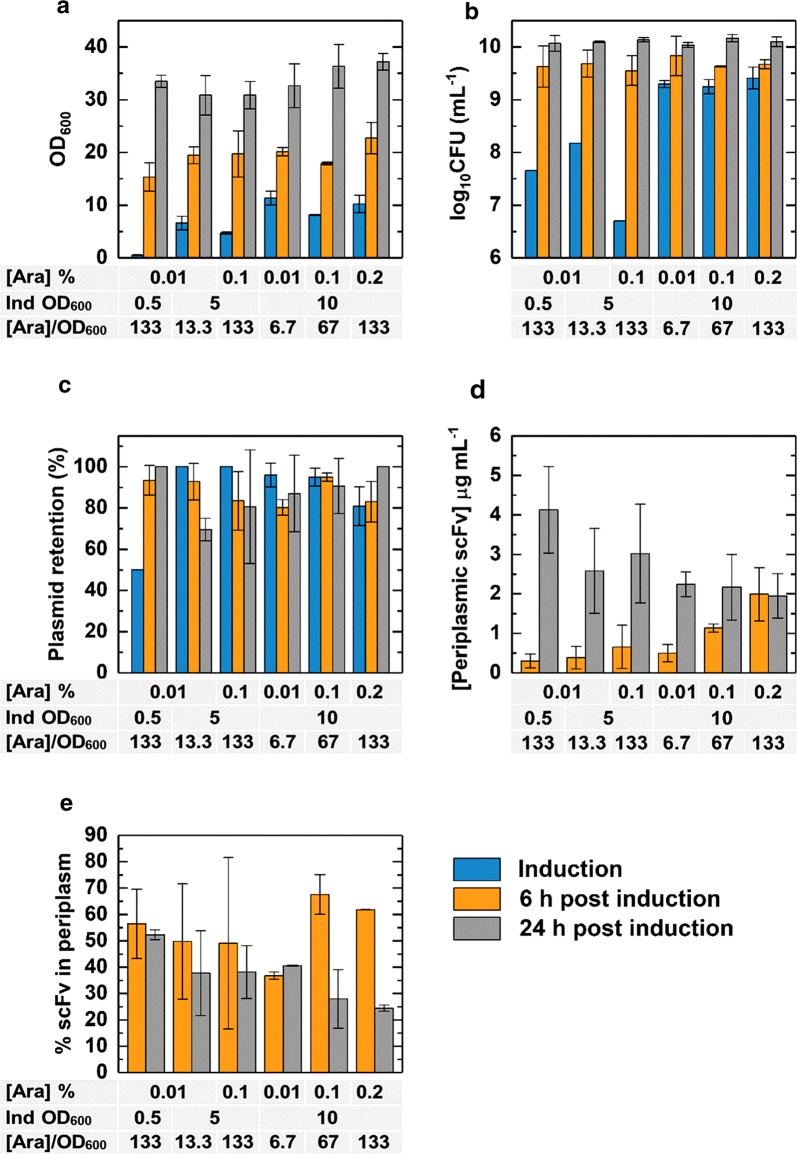
Baffled shake flask experiments to determine the effect of different induction points and inducer concentrations. Cultures were grown in 100 mL of terrific broth in 500 mL baffled shake flasks and samples were taken at induction and at 6 h and 24 h after induction. Samples were analysed for OD_600_ (**a**), CFU mL^−1^ (**b**), percentage plasmid retention (**c**), periplasmic scFv concentration (**d**) and percentage of scFv in the periplasm (**e**)

As a further optimisation step to help guide the design of future high cell density bioreactor cultures, valuable information on the growth and RPP of the PelB^sp^-scFv system at higher biomass concentrations was obtained from experiments conducted with baffled shake flasks, which deliver greater oxygen transfer and thus growth to higher optical densities (Running and Bansal 2016). First, cultures were grown at 30 °C; selected as this temperature represents an appropriate balance between productivity and solubility of scFv. Cultures were induced in early exponential (OD_600_ ≈ 0.5), mid exponential (OD_600_ ≈ 5) or late exponential (OD_600_ ≈ 10) phase, using arabinose concentrations equivalent to 133.2 µM arabinose per unit OD_600_, reflecting an equivalent number of arabinose molecules per cell (i.e. 0.01, 0.1 and 0.2% w/v arabinose for OD_600_ values of 0.5, 5 and 10 respectively). Lower arabinose concentrations were also used to induce cultures at higher OD_600_ values (Fig. 6). Cultures were sampled at the point of induction and at 6 and 24 h after induction (Fig. 6). Measurement of OD_600_ (Fig. 6a) and CFU mL^−1^ (Fig. 6b) revealed that the final biomass of cultures was not significantly affected by induction at low biomass concentrations, indicating balanced growth and RPP. Plasmid retention (Fig. 6c) remained above 80% in all but one process condition, i.e. for a low-stress culture induced with 13.3 µM arabinose per unit OD_600_ plasmid retention dropped below 70% 24 h after induction; the reason for this is unknown. For the remaining cultures, cells did not eliminate the plasmid (plasmid retained at levels of 80–100%), confirming that the energetic and metabolite requirements of growth and RPP were balanced under all other tested conditions. Analysis by flow cytometry with PI/BOX staining revealed that at least 94% of the cells in all cultures were “healthy” at all time points during growth (data not shown). At 6 h post-induction, the concentration of periplasmic scFv (Fig. 6d) was greater for cultures induced later (OD_600_ = 10) with higher concentrations of arabinose (0.2% w/v) reaching levels of 2 μg mL^−1^. The opposite trend was noted 24 h post-induction, with cultures induced at low biomass concentration yielding the highest periplasmic scFv concentration of > 4 μg mL^−1^. The proportion of scFv in the periplasm was highest (nearly 70%) in cultures induced at high biomass concentration (OD_600_ = 10) after 6 h after induction (Fig. 6e). These observations suggest that periplasmic accumulation of scFv is optimal in the hours immediately following induction of RPP, and subsequently decreases over time. The same trend was observed for production of a Fab fragment in *E. coli* (Hsu et al. 2016). It should also be noted that cultures induced at low biomass concentration are generally more variable in terms of periplasmic scFv concentration and percentage of scFv in the periplasm; highlighting the potential for increased stochasticity in exponential phase cultures.

**Fig. 7:**
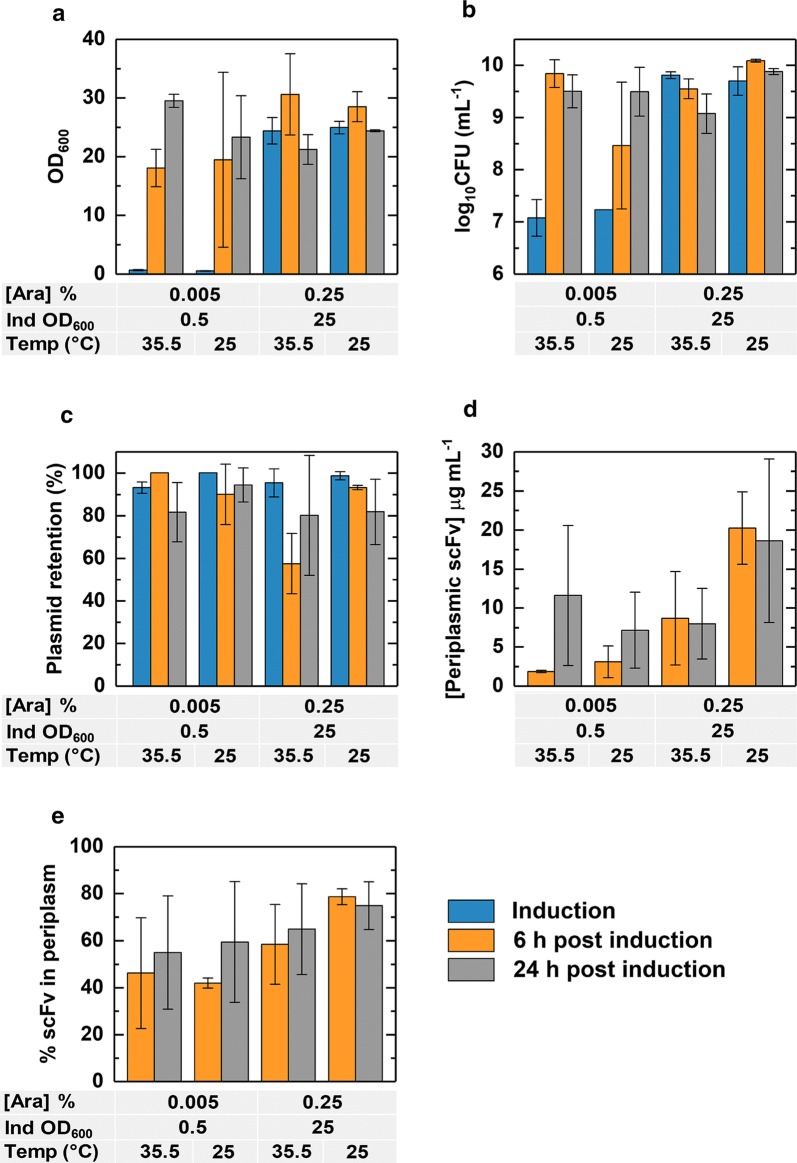
Baffled shake flask experiments to determine the effect of different growth temperatures and inducer concentrations. Cultures were grown in 100 mL of terrific broth in 500 mL baffled shake flasks and samples were taken at induction and at 6 h and 24 h after induction. Samples were analysed for OD_600_ (**a**), CFU mL^−1^ (**b**), percentage plasmid retention (**c**), periplasmic scFv concentration (**d**) and percentage of scFv in the periplasm (**e**)

We subsequently tested higher (35.5 °C) and lower (25 °C) growth temperatures, and induction at a higher biomass concentration (OD_600_ = 25) while maintaining the mid-range arabinose to biomass ratio employed above of 66.6 µM per OD_600_ unit. Measurement of OD_600_ at induction and at 6 and 24 h post induction revealed that for cultures induced at an OD_600_ of 0.5, growth was broadly comparable for both incubation temperatures (Fig. 7a). Cultures induced at an OD_600_ of around 25 showed small increases in OD_600_ at 6 h, but not after 24 h. The CFU mL^−1^ dataset revealed roughly similar trends (Fig. 7b), though small discrepancies were noted; for example for cultures induced at high biomass concentration, the lower of the two growth temperatures gave rise to higher CFU mL^−1^ values 6 and 24 h after induction despite similar values of culture OD_600_ at both temperatures (Fig. 7a). Plasmid retention (Fig. 7c) was also generally higher at lower growth temperatures, presumably due to decreased stress under such conditions.

Lower growth temperature and late induction at high biomass favoured high-level scFv production, high periplasmic concentrations (Fig. 7d) and preferential accumulation of scFv in the periplasm (Fig. 7e). The concentration of periplasmic scFv (Fig. 7d) reached maximal levels of 20 μg mL^−1^ at 25 °C 6 h after induction at high biomass concentration, and nearly 80% of the total scFv was periplasmic (Fig. 7e). The overall concentration of periplasmic scFv generated here is comparable to concentrations of Fab fragment D1.3 under stress-minimised conditions (Hsu et al. 2016); production of periplasmic Fab was likewise highest at low growth temperature.

## Discussion

The design of experiments (DoE) approach employed in this study to optimise process conditions influencing bacterial physiology and the productivity, solubility and location of scFv: (i) highlighted that titre and subcellular location of the scFv depend on the temperature and inducer concentration employed; and (ii) revealed the superiority of the PelB over the DsbA signal peptide in terms of scFv solubility and cell physiology.

For both DsbA^sp^ and PelB^sp^, temperature was the main influence on scFv concentration and solubility, with higher temperatures giving rise to increased scFv productivity, but lower scFv solubility, likely caused by an imbalance between the rates of scFv translation and translocation. Previous studies have shown that the SecYEG translocon is a bottleneck for periplasmic RPP (Schlegel et al. 2013). If the SecYEG pore becomes overloaded, both RP and native proteins are prevented from passing into the periplasm, leading to misfolding in the cytoplasm and deleterious effects on bacterial physiology. Therefore, balancing the rate of RP translation with that of translocation to the periplasm is critical. Similar results were previously observed with a Fab antibody fragment (Hsu et al. 2016).

Selection of signal peptide is known to be in important step for design of processes where RP is targeted to the periplasm (Freudl 2018); currently, in the absence of ways to accurately predict the best signal peptide to use, selection must be determined experimentally (Selas Castiñeiras et al. 2018a). In this study, whereas the DsbA signal peptide, targeting scFv to the periplasm via the co-translational SRP route, generated higher concentrations of scFv, this was at the expense of scFv solubility and bacterial physiology (membrane potential, membrane integrity and culturability). Therefore the PelB signal peptide (directing scFv to the periplasm via the post-translational SecB pathway), which gave rise to lower scFv concentrations, but far higher scFv solubility and better cell physiology, was the preferred signal peptide and was chosen as the signal peptide for further optimisation.

**Fig. 8:**
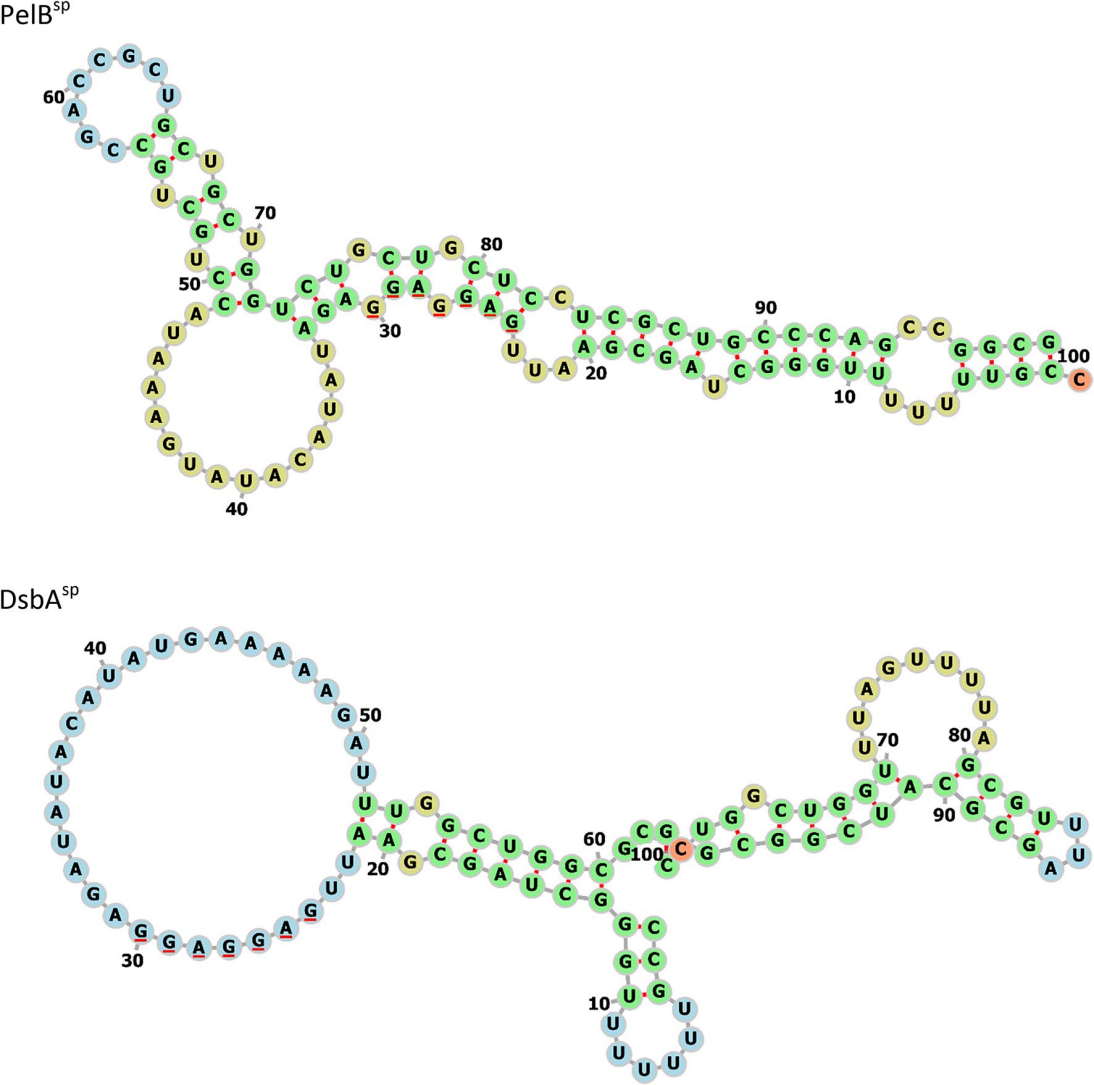
Predicted mRNA structures for the PelB^sp^ and DsbA^sp^. RNA structures were predicted using the RNAfold Webserver. Bases comprising the Shine-Dalgarno site are underlined red. Colour coding for bases: green, stems; red, multi loops at junctions; yellow, interior loops; blue, hairpin loops; orange, unpaired regions

As DsbA^sp^ targets proteins to the co-translational pathway, it is possible that large numbers of DsbA^sp^-scFv polypeptide chains sequester cellular SRP, thereby preventing SRP-mediated translocation (Bürk et al. 2009; Wickström et al. 2011), and/or blocking the Sec translocon (Schlegel et al. 2013), with consequent negative effects of cell physiology. It is known that different signal peptides can give rise to different accumulation of RPs (Selas Castiñeiras et al. 2018a), due not only to differences in rates of translocation and associated misfolding (as discussed above), but also from altered rates of translation (Heggeset et al. 2013). The structure of the mRNA encoding a RP can also influence its production and translocation to the periplasm (Ng and Sarkar 2013; Fluman et al. 2014). Prediction of the secondary structure of the mRNA transcripts encoding PelB^sp^-scFv and DsbA^sp^-scFv here reveals that the Shine-Dalgarno sequence of the DsbA^sp^-scFv mRNA is in a region of single-stranded RNA, whereas the PelB^sp^-scFv mRNA is predicted to form a structure where the Shine-Dalgarno site is located in a double-stranded region (Fig. 8). It is plausible that this could also slow down the rate of initiation of translation to account for the low overall production of the PelB^sp^-scFv observed in this study; this hypothesis will be tested in future work.

Growth at higher biomass concentration in baffled shake flasks at 25 and 35.5 °C revealed that optimal periplasmic scFv production occurred at the lower temperature and induction at a high biomass concentration (OD_600_ = 25). We envisage this information will prove useful in future work to develop bioreactor-based high cell density fermentations for scFv production. Overall, these findings confirm previous studies on stress minimisation for RPP, both for cytoplasmic (Sevastsyanovich et al. 2009; Wyre and Overton 2014; Selas Castiñeiras et al. 2018b) and periplasmic targeting (Hsu et al. 2016; Selas Castiñeiras et al. 2018a).

## Additional file

**Additional file 1: Table S1.** . List of experiments determined by central composite design. Experiments 8 to 17 represent 10 replicate runs at level ‘0’ for each factor. **Table S2.** Statistics for the model, productivity at 6 h for the DsbA^sp^-scFv system. **Table S3.** Statistics for the model, productivity at 6 h for the PelB^sp^-scFv system. **Table S4.** Statistics for the model, % solubility of scFv at 6 h for the DsbA^sp^-scFv system. **Table S5.** Statistics for the model, % solubility of scFv at 6 h for the PelB^sp^-scFv system.
