# Supplementary material for: Use of a design of experiments approach to optimise production of a recombinant antibody fragment in the periplasm of Escherichia coli: selection of signal peptide and optimal growth conditions
Source: AMB Express. 2019 Jan 7;9:5. doi: 10.1186/s13568-018-0727-8 (PMC6323050; doi:10.1186/s13568-018-0727-8)
Supplement: Supplementary file 1 — Additional file 1: Table S1. . List of experiments determined by central composite design. Experiments 8 to 17 represent 10 replicate runs at level ‘0’ for each factor. Table S2. Statistics for the model, productivity at 6 h for the DsbAsp-scFv system. Table S3. Statistics for the model, productivity at 6 h for the PelBsp-scFv system. Table S4. Statistics for the model, % solubility of scFv at 6 h for the DsbAsp-scFv system. Table S5. Statistics for the model, % solubility of scFv at 6 h for the PelBsp-scFv system. [file 13568_2018_727_MOESM1_ESM.docx]

**ADDITIONAL MATERIAL for**

**Use of a Design of Experiments approach to optimise production of a recombinant antibody fragment in the periplasm of *Escherichia coli:* selection of signal peptide and optimal growth conditions**

Ikhlaas M Kasli^1,2^, Owen RT Thomas^1^, Tim W Overton^1,2^*

^1^School of Chemical Engineering and ^2^Institute of Microbiology and Infection, University of Birmingham, Edgbaston, Birmingham, B15 2TT, UK.

*To whom correspondence should be addressed: [t.w.overton@bham.ac.uk](mailto:t.w.overton@bham.ac.uk); +44 (0) 121 414 5306

**Table S1.** List of experiments determined by central composite design. Experiments 8 to 17 represent 10 replicate runs at level '0' for each factor.

| **Experiment Number** | **Concentration of Arabinose** | | **Induction point** | | **Temperature** | |
| --- | --- | --- | --- | --- | --- | --- |
|  | **Level** | **% (w/v)** | **Level** | **OD_600_** | **Level** | **°C** |
| **1** | 0 | 0.130 | 0 | 0.75 | -α | 20.6 |
| **2** | -1 | 0.059 | -1 | 0.50 | -1 | 25.0 |
| **3** | +1 | 0.200 | -1 | 0.50 | -1 | 25.0 |
| **4** | -1 | 0.059 | +1 | 1.00 | -1 | 25.0 |
| **5** | +1 | 0.200 | +1 | 1.00 | -1 | 25.0 |
| **6** | 0 | 0.130 | -α | 0.29 | 0 | 30.3 |
| **7** | -α | 0.000 | 0 | 0.75 | 0 | 30.3 |
| **8** | 0 | 0.130 | 0 | 0.75 | 0 | 30.3 |
| **9** | 0 | 0.130 | 0 | 0.75 | 0 | 30.3 |
| **10** | 0 | 0.130 | 0 | 0.75 | 0 | 30.3 |
| **11** | 0 | 0.130 | 0 | 0.75 | 0 | 30.3 |
| **12** | 0 | 0.130 | 0 | 0.75 | 0 | 30.3 |
| **13** | 0 | 0.130 | 0 | 0.75 | 0 | 30.3 |
| **14** | 0 | 0.130 | 0 | 0.75 | 0 | 30.3 |
| **15** | 0 | 0.130 | 0 | 0.75 | 0 | 30.3 |
| **16** | 0 | 0.130 | 0 | 0.75 | 0 | 30.3 |
| **17** | 0 | 0.130 | 0 | 0.75 | 0 | 30.3 |
| **18** | +α | 0.259 | 0 | 0.75 | 0 | 30.3 |
| **19** | 0 | 0.130 | +α | 1.21 | 0 | 30.3 |
| **20** | -1 | 0.059 | -1 | 0.50 | +1 | 35.5 |
| **21** | +1 | 0.200 | -1 | 0.50 | +1 | 35.5 |
| **22** | -1 | 0.059 | +1 | 1.00 | +1 | 35.5 |
| **23** | +1 | 0.200 | +1 | 1.00 | +1 | 35.5 |
| **24** | 0 | 0.130 | 0 | 0.75 | +α | 39.9 |

**Table S2**. Statistics for the model, productivity at 6 h for the DsbA^sp^-scFv system

|  | **Sum of squares** | **df** | **Mean square** | **F value** | **p value** |  |
| --- | --- | --- | --- | --- | --- | --- |
| **Model** | 866.4354 | 9 | 96.2706 | 5.330172 | 0.0028 | significant |
| **A-[Ara]** | 0.019054 | 1 | 0.019054 | 0.001055 | 0.9745 |  |
| **B-Induction OD_600_** | 0.84512 | 1 | 0.84512 | 0.046791 | 0.8319 |  |
| **C-Temp** | 281.1835 | 1 | 281.1835 | 15.56816 | 0.0015 |  |
| **AB** | 5.326558 | 1 | 5.326558 | 0.294913 | 0.5956 |  |
| **AC** | 9.32E-01 | 1 | 9.32E-01 | 0.051609 | 0.8236 |  |
| **BC** | 30.65917 | 1 | 30.65917 | 1.697493 | 0.2136 |  |
| **A^2^** | 3.329703 | 1 | 3.329703 | 0.184354 | 0.6742 |  |
| **B^2^** | 174.9462 | 1 | 174.9462 | 9.68617 | 0.0076 |  |
| **C^2^** | 40.75301 | 1 | 40.75301 | 2.256354 | 0.1553 |  |
| **Residual** | 252.8602 | 14 | 18.06145 |  |  |  |
| **Lack of Fit** | 113.0533 | 5 | 22.61066 | 1.455549 | 0.2939 | Not significant |
| **Pure Error** | 139.8069 | 9 | 15.53411 |  |  |  |
| **Cor Total** | 1119.296 | 23 |  |  |  |  |

**Table S3.** Statistics for the model, productivity at 6 h for the PelB^sp^-scFv system

|  | **Sum of squares** | **df** | **Mean square** | **F value** | **P value** |  |
| --- | --- | --- | --- | --- | --- | --- |
| **Model** | 2.191352 | 9 | 0.243484 | 3.73335 | 0.0138 | Significant |
| **A-[Ara]** | 0.46114 | 1 | 0.46114 | 7.070691 | 0.0187 |  |
| **B-Induction OD_600_** | 0.063559 | 1 | 0.063559 | 0.974557 | 0.3403 |  |
| **C-Temp** | 0.276685 | 1 | 0.276685 | 4.242436 | 0.0585 |  |
| **AB** | 0.034142 | 1 | 0.034142 | 0.523496 | 0.4813 |  |
| **AC** | 1.21E-05 | 1 | 1.21E-05 | 0.000186 | 0.9893 |  |
| **BC** | 0.007075 | 1 | 0.007075 | 0.108485 | 0.7468 |  |
| **A^2^** | 0.020824 | 1 | 0.020824 | 0.319294 | 0.581 |  |
| **B^2^** | 0.068186 | 1 | 0.068186 | 1.045506 | 0.3239 |  |
| **C^2^** | 0.955023 | 1 | 0.955023 | 14.64344 | 0.0019 |  |
| **Residual** | 0.913059 | 14 | 0.065218 |  |  |  |
| **Lack of fit** | 0.503235 | 5 | 0.100647 | 2.210271 | 0.1422 | Not significant |
| **Pure error** | 0.409824 | 9 | 0.045536 |  |  |  |
| **Cor total** | 3.10441 | 23 |  |  |  |  |

**Table S4.** Statistics for the model, % solubility of scFv at 6 h for the DsbA^sp^-scFv system

|  | **Sum of Squares** | **df** | **Mean Square** | **F value** | **p value** |  |
| --- | --- | --- | --- | --- | --- | --- |
| **Model** | 4361.995 | 9 | 484.6661 | 4.412927 | 0.0081 | Significant |
| **A-[Ara]** | 59.65173 | 1 | 59.65173 | 0.543134 | 0.4742 |  |
| **B-Induction OD_600_** | 46.82596 | 1 | 46.82596 | 0.426355 | 0.5252 |  |
| **C-Temp** | 1172.37 | 1 | 1172.37 | 10.67454 | 0.0061 |  |
| **AB** | 0.11291 | 1 | 0.11291 | 0.001028 | 0.9749 |  |
| **AC** | 3.53E+00 | 1 | 3.53E+00 | 0.032164 | 0.8604 |  |
| **BC** | 125.528 | 1 | 125.528 | 1.142944 | 0.3045 |  |
| **A^2^** | 7.098767 | 1 | 7.098767 | 0.064635 | 0.8033 |  |
| **B^2^** | 67.59478 | 1 | 67.59478 | 0.615456 | 0.4468 |  |
| **C^2^** | 2006.189 | 1 | 2006.189 | 18.26653 | 0.0009 |  |
| **Residual** | 1427.773 | 13 | 109.8287 |  |  |  |
| **Lack of Fit** | 631.8882 | 4 | 157.9721 | 1.786375 | 0.2157 | Not significant |
| **Pure Error** | 795.8848 | 9 | 88.43165 |  |  |  |
| **Cor Total** | 5789.768 | 22 |  |  |  |  |

**Table S5.** Statistics for the model, % solubility of scFv at 6 h for the PelB^sp^-scFv system

|  | **Sum of Squares** | **df** | **Mean Square** | **F value** | **p value** |  |
| --- | --- | --- | --- | --- | --- | --- |
| **Model** | 239.5322 | 3 | 79.84408 | 3.170124 | 0.0468 | significant |
| **A-[Ara]** | 28.98538 | 1 | 28.98538 | 1.150833 | 0.2961 |  |
| **B-Induction OD_600_** | 0.178765 | 1 | 0.178765 | 0.007098 | 0.9337 |  |
| **C-Temp** | 210.3681 | 1 | 210.3681 | 8.35244 | 0.0091 |  |
| **Residual** | 503.7285 | 20 | 25.18642 |  |  |  |
| **Lack of Fit** | 2.58E+02 | 11 | 2.35E+01 | 0.859192 | 0.6006 | Not significant |
| **Pure Error** | 245.7064 | 9 | 27.30071 |  |  |  |
| **CorTotal** | 743.2607 | 23 |  |  |  |  |
